# ﻿Comparative mitogenomic analysis reveals variations and evolution of ectomycorrhizal fungal *Strobilomyces*

**DOI:** 10.3897/imafungus.16.141848

**Published:** 2025-02-17

**Authors:** Chao Liu, Wan-Ying Li, Le-Xuan Zheng, Mi Dao, Huan-Huan Chen, Li-Hong Han

**Affiliations:** 1 College of Biological Resource and Food Engineering, Yunnan Engineering Research Center of Fruit Wine, Qujing Normal University, Qujing, Yunnan, 655011, China Qujing Normal University Qujing China

**Keywords:** *Boletales*, mitogenome, gene re-arrangement, phylogenomics

## Abstract

The genus *Strobilomyces*, representing a diverse and widespread group of ectomycorrhizal mushroom-forming fungi, plays a crucial ecological and economical role. However, until now, a comprehensive description of its mitochondrial genome (mitogenome) has been lacking. In our current study, we have successfully assembled and analysed the mitogenomes of five *Strobilomyces* species. These mitogenomes span a range from 35,618 base pairs (bp) to 42,088 bp, exhibiting a higher nucleotide abundance of AT compared to GC. All five mitogenomes harbour 14 conserved protein-coding genes (PCGs), two ribosomal RNAs (rRNAs) and 24 transfer RNAs (tRNAs). Notably, the overall ratio of Ka/Ks for all PCGs was found to be less than 1.0, indicating that these genes have undergone purifying selection during evolution. Intriguingly, the mitogenomic comparison revealed two instances of gene re-arrangement, which were directly linked to the geographical distribution of the *Strobilomyces* species. The concatenated mitochondrial PCGs (mtPCGs) and nuclear ribosomal DNA (nrDNA) phylogenies displayed a robust congruent topology at the family level. Specifically, the *Strobilomyces* species clustered together and formed sister relationship with other *Boletaceae* species in the mtPCGs tree. In contrast, the *Strobilomyces* species grouped at the base of the nrDNA tree when concerning *Boletaceae*. This study represents the first report on the mitogenomes of the *Strobilomyces* genus, providing valuable insights into fungal evolution within *Boletales*.

## ﻿Introduction

The mitochondrion, a functionally crucial organelle in fungi, harbours its own genetic material (Butenko et al. 2024). Unlike nuclear genomes, fungal mitochondrial genomes (mitogenomes) are typically inherited uniparentally and undergo rapid evolution at the nucleotide sequence level. Mitogenomes have been instrumental in understanding phylogenetic evolution and population genetics (Yu et al. 2023b). Comparative analyses of fungal mitogenome sequences have revealed that, despite general conservation of gene content, gene order varies both within and between taxa. These variations and evolutionary patterns in the mitogenome have become important molecular markers for resolving evolutionary relationships in fungi (Li et al. 2022). Currently, complete mitogenome sequences are available for species representing 15 genera within *Boletales* (Li et al. 2021). However, the mitogenome of *Strobilomyces* species remains unexplored.

*Strobilomyces* Berk. (*Boletaceae*, *Boletales*, *Agaricomycetes*) comprises over 40 species (http://www.indexfungorum.org/), predominantly distributed in subtropical and tropical regions of Asia and Africa (Han et al. 2020). Members of this genus are characterised by their shaggy to scaly, blackish or greyish appearance, partial veils, woolly stems and flesh that discolours to reddish when sliced, eventually turning black (Han et al. 2020). These fungi form ectomycorrhizal associations with hardwood trees, establishing mutualistic relationships with their host plant families (Han et al. 2018). Previous studies have attempted to clarify species taxonomy and systematic evolution within *Strobilomyces* using nuclear and mitochondrial DNA markers (Sato and Murakami 2009; Gelardi et al. 2013; Han et al. 2018; Han et al. 2019; Han et al. 2020). However, the current taxonomy of *Strobilomyces* is somewhat confusing, especially considering the numerous new species reported in the past two decades (Han et al. 2020; Deng et al. 2023).

Due to the abundance of molecular markers present in mitogenomes and their unique evolutionary trajectories, these genomes are highly sought-after tools for reconstructing phylogenetic relationships (Li et al. 2019; Christinaki et al. 2022; Li et al. 2022; Zhong et al. 2022). In order to recognise the pivotal role of the mitogenome in elucidating the phylogenetics and adaptive evolution of *Boletales*, this study aims to: (1) uncover the genetic characteristics of *Strobilomyces* mitogenomes; (2) compare the mitogenomes within the genus *Strobilomyces*; and (3) determine the phylogenetic position of *Strobilomyces* within *Boletales*, based on a combined dataset of mitochondrial genes encompassing 14 core protein-coding genes (PCGs). This research represents the first report on the mitogenomes of *Strobilomyces* species. Our study aims to enhance the understanding of the structural and evolutionary dynamics of mitogenomes in *Strobilomyces* species, as well as their phylogenetic affiliations with closely-related fungal taxa.

## ﻿Materials and methods

### ﻿Sample information

*Strobilomycesalpinus* strain H2022S10 was collected from Pudacuo Nature Reserve in Shangri-La, Yunnan, China, on 6 August 2022. The specimens were collected with the utmost care and stored at ultra-low temperature freezer at -80 °C. Identification was conducted, based on a combination of morphological characteristics and multigene phylogenetic analysis by Li-Hong Han. The specimen has been deposited in the Herbarium of the College of Biological Resources and Food Engineering at Qujing Normal University, under the accession number H2022S10.

Additionally, four unassembled sequencing datasets of *Strobilomyces* species were retrieved from the Sequence Read Archive database. The four species include *S.echinocephalus* from China (accession number SRR28540251), an unidentified species, *Strobilomyces* sp. 1 (SRR28540241) from the USA, *S.confusus* from Vietnam (SRR28540078) and another unidentified species, *Strobilomyces* sp. 2 (SRR28540220) from Vietnam (Tremble et al. 2024).

### ﻿Mitogenome sequencing, assembly and annotation

Whole-genomic DNA was extracted from the unpolluted context of the stipe using the CTAB method (Doyle and Dickson 1987). Libraries were constructed by MGIEasy Universal DNA Library Prep Kit v.1.0, following the standard protocol. Briefly, 1 μg of genomic DNA was randomly fragmented by Covaris and fragments averaging 200–400 bp in size were selected with MGIEasy DNA Clean beads. Subsequently, the fragments were end-repaired, 3’ adenylated and ligated with adapters. The DNA samples were amplified via PCR and the products were purified by the MGIEasy DNA Clean beads. The double stranded PCR products were heat denatured and circularised by the splint oligo sequence in MGIEasy Circularisation Module. The single strand circular DNA was formatted as the final library and assessed for quality control. Sequencing was then performed on a DNBSEQ-T7RS platform, generating 150 bp paired-end reads by GrandOmics Biosciences Co., Ltd. (Wuhan, China), yielding over 5.0 GB of raw data. The mitogenome sequences were assembled using GetOrganelle v.1.7.5 (Jin et al. 2020) with the filtered data. Annotation of the mitogenomes was primarily carried out using GeSeq (Tillich et al. 2017) and MITOS (http://mitos.bioinf.uni-leipzig.de/index.py) , following the mould mitochondrial genetic code, with manual corrections made where necessary. The graphical representation of the mitogenome was visualised using Chloroplot (Zheng et al. 2020).

### ﻿Sequence analysis of *Strobilomyces* mitogenomes

The strand asymmetries in the five *Strobilomyces* mitogenomes were evaluated using the formulae: AT skew = [A - T] / [A + T] and GC skew = [G - C] / [G + C]. The codon usage frequency and preference in the *S.alpinus* mitogenome were analysed using the Sequence Manipulation Suite (https://detaibio.com/sms2/index.html), based on genetic code 4. The RNA editing sites in 15 PCGs of *S.alpinus* were predicted using the PmtREP programme (http://112.86.217.82:9929/#/tool/alltool/detail/336), with a threshold value of 0.2. The pairwise genetic distances amongst the 14 core PCGs, excluding *rps3* due to incompleteness in the mitogenomes *Strobilomyces* sp. 1 and *S.echinocephalus*, were calculated using the Kimura-2-parameter (K2P) substitution model in MEGA X (Kumar et al. 2018). Furthermore, the non-synonymous (Ka) and synonymous (Ks) substitution rates for the core PCGs in the five *Strobilomyces* mitogenomes were estimated using DnaSP v.6 (Rozas et al. 2017). To identify simple sequence repeats (SSRs) within the mitogenomes, we utilised the MISA programme (Beier et al. 2017) with predefined thresholds for the minimum number of repeats: 10 for mononucleotides, five for dinucleotides, four for trinucleotides and three for tetranucleotides, pentanucleotides and hexanucleotides. For the detection of tandem repeats, we employed Tandem Repeats Finder v.4.09 software (Benson 1999) with default parameters. Additionally, we identified forward, reverse, palindromic and complementary repeats based on REPuter (Kurtz et al. 2001). We set the maximum number of computed repeats to 5,000, the minimum repeat size to 30 and the Hamming distance to 3.

### ﻿Comparison of *Strobilomyces* mitogenomes

To gain insights into the evolution of mitogenomes in *Strobilomyces*, we conducted a comparative analysis of five mitogenomes. This analysis included evaluating genome size, gene content, gene order, genetic distance and selection pressure. For a more detailed visualisation of syntenies and potential gene re-arrangement events, we performed a mitogenome collinearity analysis using Mauve (Stajich et al. 2010).

### ﻿Phylogenetic analysis

To determine the phylogenetic position of *Strobilomyces* within *Boletales*, we assembled two datasets based on nucleotide sequences. The first dataset comprised concatenated sequences of 14 core PCGs from mitogenome, excluding the *rps3* gene due to incompleteness in two of the mitogenomes. The second dataset consisted of nuclear ribosomal DNA (nrDNA) sequences, specifically ETS-18S-ITS1-5.8S-ITS2-26S. We utilised 36 representative mitogenomes and 85 nrDNA sequences from *Boletales* taxa, along with two outgroup species from *Russulales*: *Russulacompacta* and *Lactariusdeliciosus* (Suppl. material 1: table S1). Sequence alignment was performed using MAFFT (Katoh et al. 2019) with default parameters. Phylogenetic relationships were inferred by both Maximum Likelihood (ML) and Bayesian Inference (BI) approaches, with parameters specified in our previous study (Yu et al. 2023a). The GTR+F+R3 substitution model was selected for the mtPCGs dataset, while the GTR+F+R8 model was applied to the nrDNA trees. To further assess the phylogenetic topology, we employed the BI method in MrBayes v.3.1.2 (Ronquist et al. 2012). Four Markov Chain Monte Carlo (MCMC) chains were run simultaneously for 20 million generations, sampling trees every 100 generations. In evaluating phylogenetic confidence, we considered nodes to be strongly supported by both ML bootstrap values (MLB ≥ 70%) and Bayesian posterior probabilities (BPP ≥ 0.95).

### ﻿Abbreviations

**BI** Bayesian Inference

**K2P** Kimura-2-parameter

**Ka** Non-synonymous substitution rate

**Ks** Synonymous substitution rate

**Mitogenome** Mitochondrial genome

**ML** Maximum Likelihood

**nrDNA** Nuclear ribosomal DNA

**PCG** Protein-coding gene

**RSCU** Relative synonymous codon usage

**SSRs** Simple sequence repeats

## ﻿Results

### ﻿Characterisation of the *Strobilomyces* mitogenomes

The five *Strobilomyces* species exhibited complete mitogenome sequences ranging from 35,618 bp in *S.* sp. 2 to 42,088 bp in *S.alpinus* (Table 1). The comparative analysis indicated that all five mitogenomes possessed circular DNA molecules. The nucleotide composition revealed a higher abundance of AT (77.3%) compared to GC (22.7%), with 37.7% A, 39.5% T, 11.7% G and 11.0% C. The negative AT skew and positive GC skew (except in *S.* sp. 1) indicated a higher frequency of T and G over A and C in the forward strand, respectively, which is consistent with the overall nucleotide ratio.

**Figure 1.**
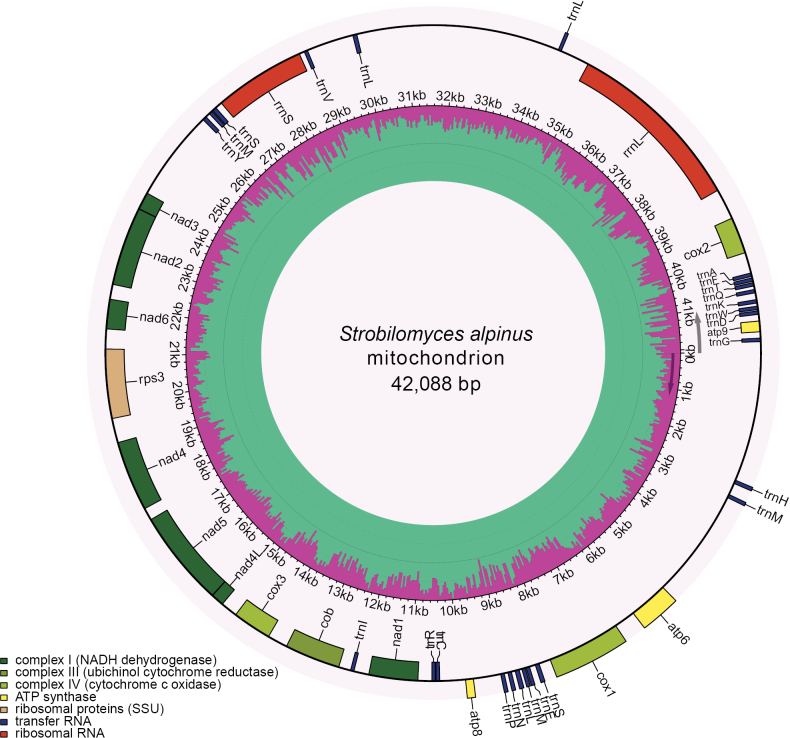
Circular map of the mitogenome of *S.alpinus*, with genes colour-coding according to their functional groups. The inner ring illustrates the GC content.

The mitogenomes encoded 14 core PCGs essential for energy metabolism. These include three ATP synthases, three cytochrome c oxidases, seven NADH dehydrogenases and one ubiquinol cytochrome c reductase. Additionally, the gene *rps3* encodes a ribosomal protein, which is involved in translation, along with two rRNAs and 24 tRNAs, were identified. In the *S.alpinus* mitogenome, most genes—excluding *atp6*, *atp8*, *cox1* and nine tRNA genes—are transcribed on the forward strand (Fig. 1).

**Table 1.** General features of mitogenomes from five *Strobilomyces* species.

Characteristic	*S.alpinus*	*S.confusus*	*S.* sp. 1	*S.echinocephalus*	*S.* sp. 2
Accession number	C_AA098151.1	SRR28540078	SRR28540241	SRR28540251	SRR28540220
Size (bp)	42088	36070	36572	38099	35618
A (%)	37.64	37.75	37.26	38.39	37.64
T (%)	39.86	39.46	39.67	39.04	39.61
C (%)	10.83	10.88	11.69	10.74	10.84
G (%)	11.66	11.9	11.37	11.83	11.91
AT (%)	77.5	77.22	76.94	77.43	77.25
GC (%)	22.5	22.78	23.06	22.57	22.75
AT-skew	-0.03	-0.02	-0.03	-0.01	-0.03
GC-skew	0.04	0.04	-0.01	0.05	0.05
PCGs	15	15	15	15	15
rRNA	2	2	2	2	2
tRNA	24	24	24	24	24

### ﻿Codon usage analysis of PCGs

Amongst the amino acids found in the mitogenomes of *Strobilomyces* species, leucine, isoleucine and serine were the most frequently utilised. Specifically, leucine (14.8%) was the most prevalent, followed by isoleucine (12.1%) and serine (8.71%). Cysteine (0.6%) and tryptophan (1.1%) were the least frequently encountered residues (Suppl. material 2: fig. S1). Notably, the codon usage patterns exhibited remarkable consistency across the *Strobilomyces* species.

For further analysis, we focused on the mitogenome of *S.alpinus* to compare the distribution and frequency of codon usage. All core PCGs and the ribosomal protein-coding gene *rps3* in the *S.alpinus* mitogenome possessed the canonical AUG start codon and UAA stop codon. However, in the *atp8* and *nad4L* genes from two Vietnamese samples (*S.confusus* and *S.* sp. 2), the UAG codon functioned as the stop codon.

**Figure 2.**
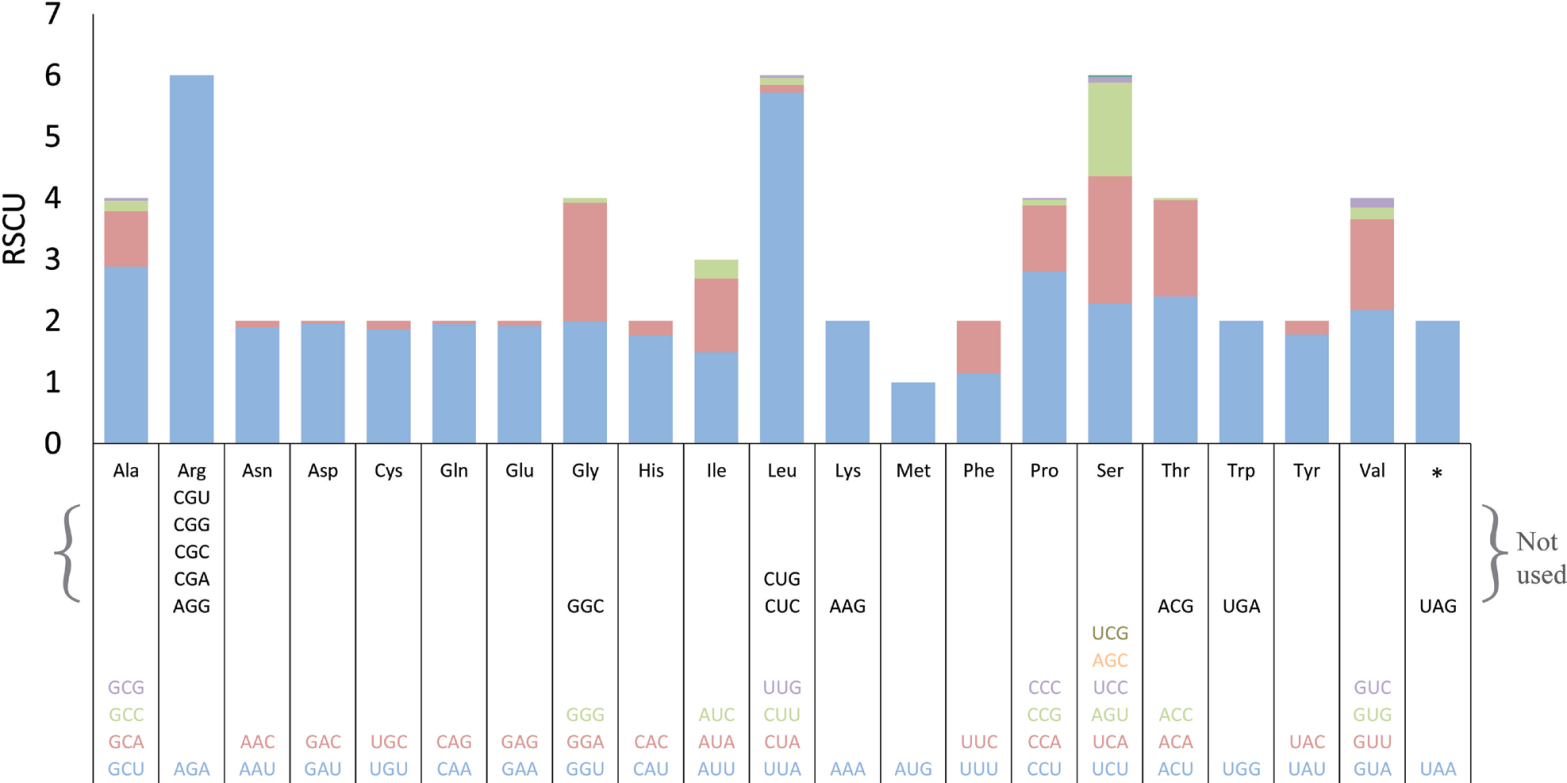
Codon usage for 15 PCGs in the mitogenome of *S.alpinus*. The 15 PCGs consist of 14 typical PCGs and *rps3*.

The relative synonymous codon usage (RSCU) was calculated for the 15 PCGs in the mitogenome of *S.alpinus*. The RSCU values of 27 codons exceeded 1.00, with 14 codons ending in U, 12 ending in A and only one ending in G (Fig. 2). This observation indicates a strong bias towards NNA and NNT codons when available. Amongst these preferred codons, despite arginine being encoded by six different codons, only AGA (with an RSCU value 6.0) was utilised to encode this amino acid. The codon for leucine (UUA) was the second most preferred, with an RSCU value of 5.7. Seven codons (AGG, CGA, CGC, CGG, CGU, CUC and UGA) were never used, while five codons (GGC, CUG, AAG, ACG and UAG) were used in some *Strobilomyces* species at a very low frequency. Notably, the tryptophan codon UGA, which functions as a stop codon in the standard genetic code, was not utilised in the mitogenome of *S.alpinus*.

### ﻿Prediction of RNA editing sites in PCGs

**Figure 3.**
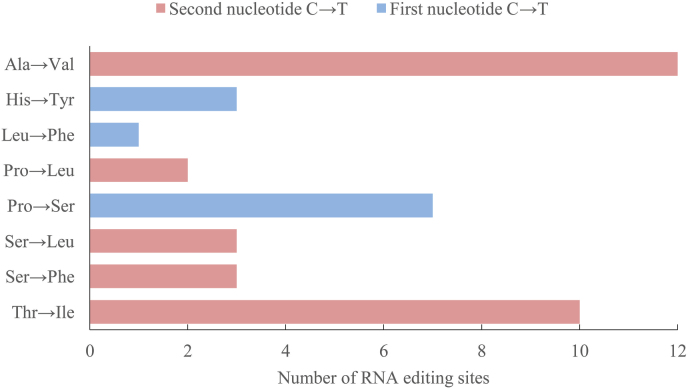
Prediction of RNA editing site numbers and amino acid changes.

In our analysis of the mitogenome of *S.alpinus*, we predicted 41 RNA editing sites that involve the conversion of cytosine (C) to uridine (U) within 12 of the 15 PCGs (Fig. 3). Notably, no RNA editing was observed in three genes: *atp6*, *atp8* and *nad4L*. The frequency of these editing events varied significantly amongst the PCGs. The *nad5* gene exhibited the highest number of RNA editing sites (7), followed by the *nad2* and *nad4* genes, each with six sites. Amongst these editing sites, 26.8% (11) were located at the first position of the codon triplet, while 73.2% (30) occurred at the second position.

After RNA editing, the hydrophobicity of 43.9% of the amino acids remained unchanged. However, 39.0% of the amino acids were predicted to transition from hydrophilic to hydrophobic, while 17.1% were predicted to change from hydrophobic to hydrophilic. The most frequent amino acid substitution, occurring 12 times, was from alanine to valine, while the rarest substitution was from leucine to phenylalanine, occurring only once amongst all editing events.

### ﻿Genetic distance and evolutionary rates of core genes

**Figure 4.**
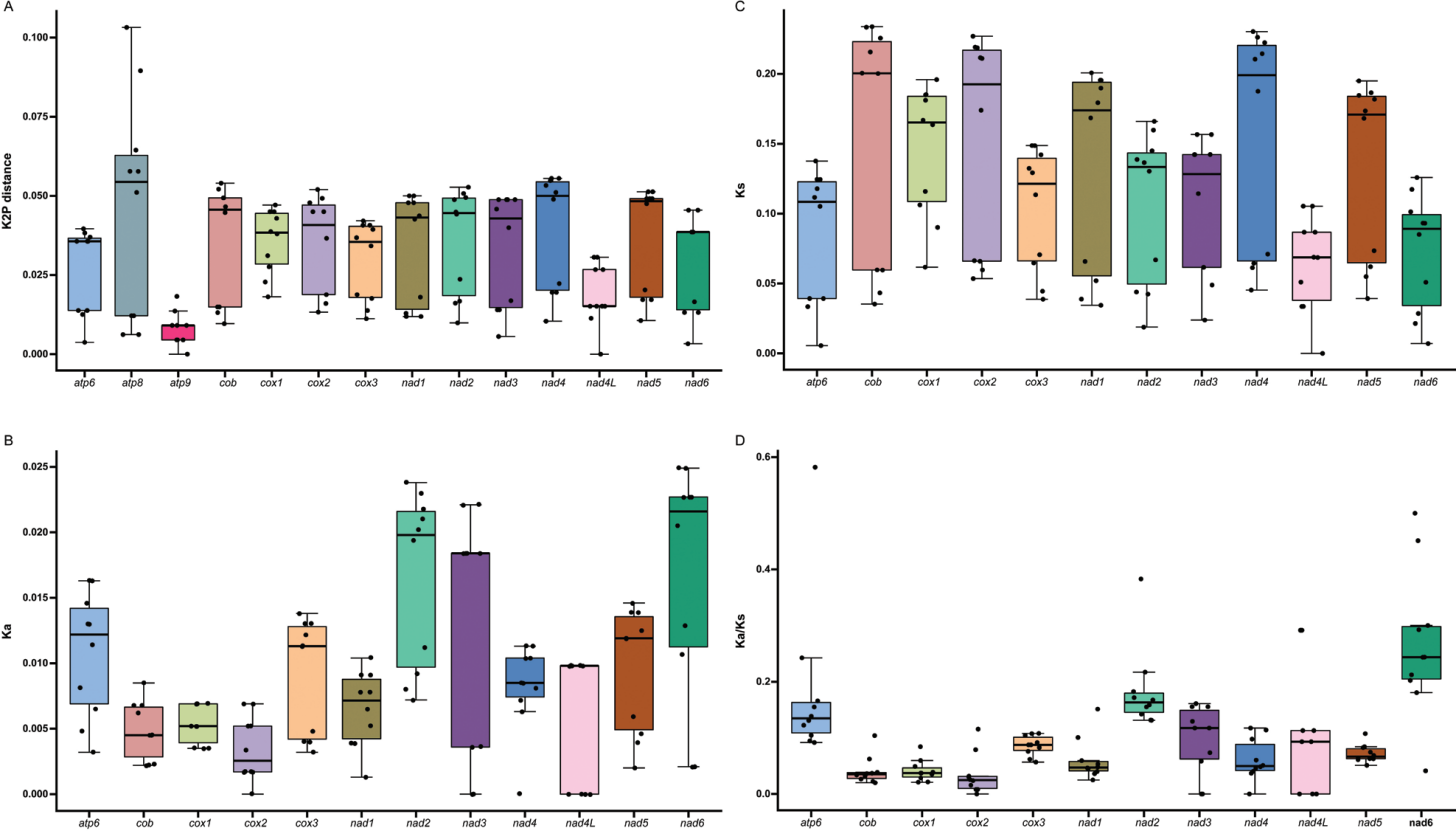
Genetic and evolutionary analyses of core genes in the five *Strobilomyces* mitogenomes. **A** Kimura-2-parameter (K2P) distance, **B** number of non-synonymous substitutions per non-synonymous site (Ka), **C** number of synonymous substitutions per synonymous site (Ks), **D**Ka/Ks ratio.

As the *rps3* gene was frequently absent or incomplete in the mitogenomes, we focused on 14 core PCG genes: *atp6*, *atp8*, *atp9*, *cob*, *cox1*, *cox2*, *cox3*, *nad1*, *nad2*, *nad3*, *nad4*, *nad5*, *nad6* and *nad4L* to calculate genetic distances and substitution rates amongst the five *Strobilomyces* species. Our results showed that the genetic distance ranged from 0.01 (*atp9*) to 0.05 (*atp8*) based on the K2P model (Fig. 4A), demonstrating a high degree of conservation amongst these genes. Due to multiple instances of zero Ka or Ks values in *atp8* and *atp9*, only 12 remaining genes were included in the analysis. Amongst these 12 genes, the *nad6* gene exhibited the highest Ka value, followed by *nad2*, while *cox2* showed the lowest Ka value (Fig. 4B). The *nad4* gene had the highest Ks value, whereas *nad4L* displayed the lowest Ks value (Fig. 4C). The overall ratio of Ka/Ks for all detected genes was below one, ranging from 0.03 (*cox2*) to 0.27 (*nad6*) (Fig. 4D). This finding indicates that these genes have undergone purifying selection throughout their evolution. It is important to note that when comparing Ka/Ks ratios, three genes—*nad6*, *nad2* and *atp6*—exhibited significantly higher ratios (Ka/Ks > 0.1), while three other genes—*cox2*, *cob* and *cox1*—showed notably lower ratios (Ka/Ks < 0.05).

**Figure 5.**
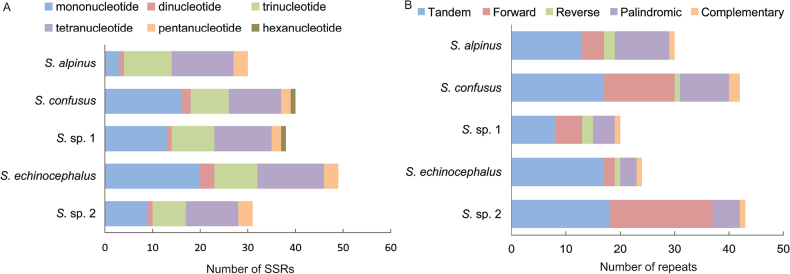
Number of SSRs (**A**) and long repeats (**B**) in the five *Strobilomyces* mitogenomes.

### ﻿Repeat sequences analysis

To gain a deeper understanding of the characteristics of repeat sequences, we identified SSRs, tandem repeats and dispersed repeats within the mitogenomes of *Strobilomyces* in this study. Our results showed that the number of SSRs detected ranged from 30 in *S.alpinus* to 49 in *S.echinocephalus* (Suppl. material 1: table S2 and Fig. 5A). Generally, mononucleotide and tetranucleotide repeats were the most prevalent, followed by trinucleotide repeats, while hexanucleotide repeats were the least common. Mononucleotide repeats of A/T were found to be the most abundant type of repeat. Notably, only three instances of A/T repeats, each consisting of 10 nucleotides, were found in *S.alpinus*. Mononucleotide repeats of C/G were exceedingly rare, with only one instance of an 11-nucleotide repeat identified in *S.* sp. 2. Dinucleotide repeats of AT/AT were detected between one and three times across the *Strobilomyces* mitogenomes, while no AG/CT repeats were observed. It is intriguing to note that, despite possessing the largest mitogenome (42,088 bp) amongst the studied species, *S.alpinus* had the fewest SSRs.

The analysis of interspersed repetitive sequences revealed an uneven distribution amongst the *Strobilomyces* mitogenomes (Fig. 5B). The number of repeats ranged from 7 in *S.echinocephalus* to 25 in both *S.confusus* and *S.* sp. 2. Forward repeats, which ranged from 2 to 19, were the most common type, followed by palindromic repeats, ranging from 3 to 10. Reverse and complementary repeats occurred one to two times in each mitogenome. Notably, 91% of the repetitive sequences were between 30 and 49 bp in length, while only 9% ranged from 50 to 80 bp.

Furthermore, the analysis of tandem repeats revealed their presence in each mitogenome, with quantities ranging from 8 to 18 varying between 25 and 86 bp (Suppl. material 1: table S3 and Fig. 3). Most of these tandem repeats consisted of two copies, with 71% measuring between 25 and 50 bp and 29% measuring between 51 and 86 bp (Suppl. material 1: table S3). Interestingly, the number of tandem repeats did not correlate with the size of the mitogenome.

### ﻿Comparison of the mitogenomes of *Strobilomyces* species

**Figure 6.**
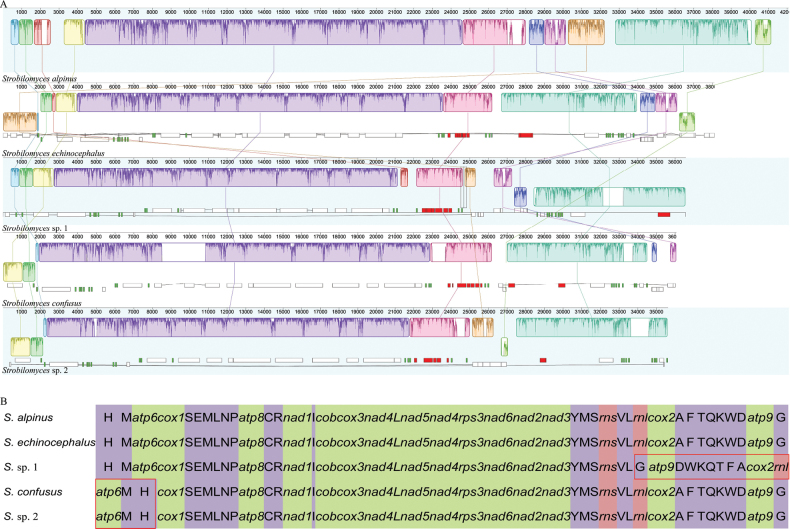
Collinearity (**A**) and gene order comparison (**B**) amongst five *Strobilomyces* mitogenomes. Genes (green), rRNA (orange) and tRNA (purple) are represented in their respective colours, while gene re-arrangements across the mitogenomes are indicated by red boxes.

To conduct an intensive study of the evolution of *Strobilomyces* mitogenomes, a comparative analysis was performed amongst five species. The Mauve alignment highlighted a conserved synteny amongst the mitogenomes, which were organised into 11 homologous regions represented by distinct coloured synteny blocks (Fig. 6A). This alignment revealed a high degree of synteny across the mitogenomes. However, two instances of gene re-arrangement were identified. Specifically, the gene linkage comprising *trnG*, *atp9*, *trnD*, *trnW*, *trnK*, *trnQ*, *trnT*, *trnF*, *trnA*, *cox2* and *rnl* underwent a reverse re-arrangement in the mitogenome of *Strobilomyces* sp. 1 from the USA. Another re-arrangement, involving the gene linkage of *trnH*, *trnM* and *atp6*, was observed in two mitogenomes—*S.confusus* and *Strobilomyces* sp. 2—both originating from Vietnam (Fig. 6B).

### ﻿Phylogenetic analyses

**Figure 7.**
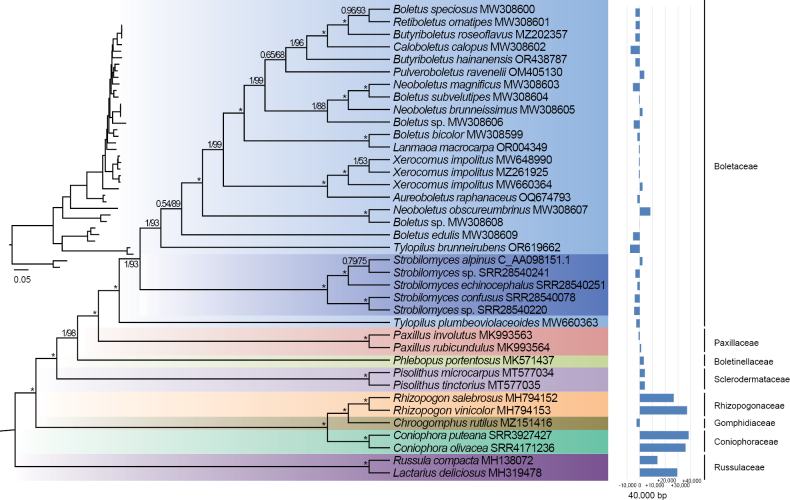
Phylogenetic analysis of *Boletales* species, based on concatenated nucleotide sequences of 14 typical mtPCGs. The tree presented here is the single best topology recovered from Maximum Likelihood analysis. Support values for the nodes are indicated as ML/BI with an asterisk (*) denoting a value of 1 or 100. The phylogenetic clades are colour-coded at the family level, with dark blue representing the genus *Strobilomyces*.

**Figure 8.**
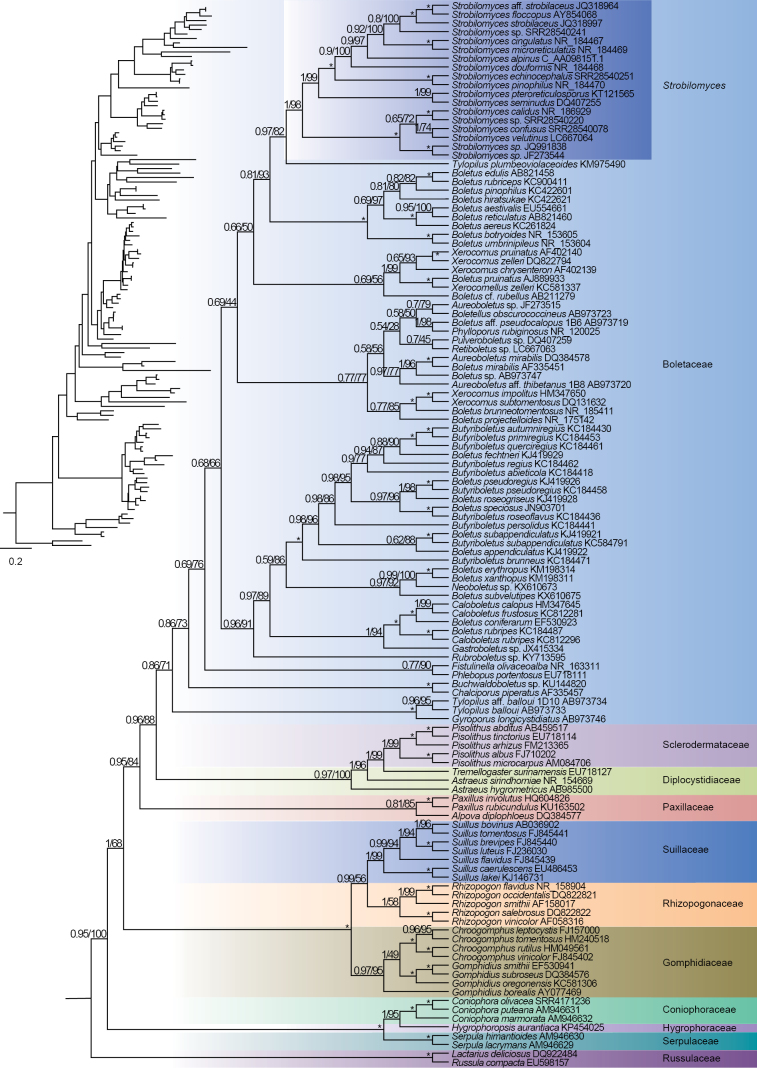
Phylogenetic relationships amongst representative specimens of *Boletaceae*, inferred from the nrDNA dataset using Maximum Likelihood and Bayesian Inference methods (only the ML tree is displayed). The phylogenetic clades are distinguished by different colours at the family level, with dark blue representing the genus *Strobilomyces*.

Two phylogenetic construction methods, BI and ML, produced consistent topological structures. However, the phylogenetic trees constructed from different datasets, specifically mtPCGs and nrDNA, exhibited distinct topologies (Figs 7, 8). The summary coalescent tree revealed major nodes with substantial statistical support.

Overall, the concatenated mtPCGs and nrDNA phylogenies exhibited congruent topologies at the family level, with a few exceptions. Species within the same family clustered together, including *Russulaceae*, *Coniophoraceae*, *Gomphidiaceae*, *Rhizopogonaceae*, *Sclerodermataceae*, *Paxillaceae* and *Boletaceae*. One notable exception was the family *Boletinellaceae*, which formed a monophyletic clade in the phylogeny of mtPCGs, but was integrated into the *Boletaceae* family in the phylogeny of nrDNA. The phylogenetic relationships at the family level also revealed subtle differences between the mtPCGs and nrDNA trees (Figs 7, 8). For instance, *Strobilomyces* species clustered together to form a sister branch alongside other *Boletaceae* species in the mtPCGs tree. In contrast, in the nrDNA tree, the *Strobilomyces* species were positioned at the base of *Boletaceae*. *Boletaceae* represents a complex clade characterised by conflicting phylogenetic markers. Despite this, fungal species within each family clustered together with high support values (Fig. 7).

Many of the recovered groups within the *Boletales* are consistent with the previous studies (Tremble et al. 2024). In our analysis of the mitogenome sizes, we found that the families situated at the base of the phylogenetic tree tend to possess larger mitogenomes. As species evolve, the size of their mitochondria generally decreases.

## ﻿Discussion

The *Strobilomyces* species possess significant ecological and medicinal values, forming ectomycorrhizal associations with families such as *Casuarinaceae*, *Dipterocarpaceae*, *Fabaceae*, *Fagaceae*, *Myrtaceae* and *Pinaceae* (Han et al. 2020). Notably, certain *Strobilomyces* species are recognised for their edible and anticancer properties, placing them on the list of Chinese edible mushrooms (Dai and Yang 2008). In the current study, we report the mitogenomes of *Strobilomyces* species for the first time, revealing similarities in genome size and gene content amongst them.

The complete mitogenomes of the sampled *Strobilomyces* species range from 35,618 bp to 42,088 bp. These sizes are comparable to those of *Boletus* (Li et al. 2021), *Cordyceps* (Zhang et al. 2023) and *Phytophthora* (Winkworth et al. 2022), but are smaller than those of *Ganoderma* (Li et al. 2022), *Amanita* (Li et al. 2020), *Tricholoma* (Huang et al. 2021) and *Ophiocordyceps* (Zhong et al. 2022). Typically, these mitogenomes contain 14 core PCGs, 2 rRNA genes and 24 tRNA genes, with only a few exceptions regarding partial genes.

Consistent with previous studies, the nucleotide composition of the *Strobilomyces* mitogenomes exhibits a high AT content of approximately 77%. This is similar to that of *Boletus* (Li et al. 2021) and *Tricholoma* (Huang et al. 2021), but higher than that of *Ganoderma* (Li et al. 2022) and *Cordyceps* (Zhang et al. 2023), while being lower than that of *Trametes* (Chen et al. 2021).

The analysis of codon usage patterns in fungal mitogenomes is essential for understanding the fundamentals of molecular biology (Arella et al. 2021). These patterns reflect variations in local base compositional biases and the influence of natural selection. While codon usage tends to be conserved at the genus level, more significant variations are observed when comparing lineages at a more distant level. Notably, fungal mitochondrial genes exhibit a preference for AU-rich codons, as previously reported in studies such as Zhang et al. (2023). Consistent with earlier research on fungi (Zhong et al. 2022; Zhang et al. 2023), the mitogenomes of *Strobilomyces* species displayed a pattern in which several GC-rich codons were never utilised. However, codon usage was significantly more diverse in *Ganoderma* (Li et al. 2022) and *Tricholoma* (Huang et al. 2021). In the closely-related genus *Boletus*, Zhang et al. (2023) reported a varied codon usage that warrants further investigation.

RNA editing, a prevalent phenomenon in fungal mitogenomes, plays a crucial role in protein folding and the expression of mitochondrial genes (Teichert 2020). Specifically, the conversion of cytosine to uridine alters genomic information, enhancing protein stability in fungi by modifying codons (Kirti et al. 2023). In *S.alpinus*, 73.2% of the 41 RNA editing sites occurred at the second codon position, while the remaining sites were located at the first codon position. Furthermore, 39.0% of the amino acids were predicted to change from hydrophilic to hydrophobic, including conversions from threonine to isoleucine, serine to phenylalanine and serine to leucine. Notably, the number of conversions from polar to non-polar and from hydrophilic to hydrophobic amino acids exceeded those in the opposite direction. The identification of these RNA editing sites offers essential insights for predicting gene functions associated with newly-introduced codons (Cheng et al. 2021).

Repeat sequences, a distinguishing feature of fungal mitogenomes, exhibit high polymorphism and widespread occurrence, significantly contributing to genomic structural variations (Zhong et al. 2022). These sequences primarily manifest as SSRs, tandem repeats and dispersed repeats. Notably, the mitogenome of *Strobilomyces* species contains a substantial proportion of repeat sequences. Amongst the genes analysed, *atp9* exhibited the lowest mean K2P genetic distance, indicating a high degree of conservation across the mitogenomes. Intriguingly, *nad6* displayed the highest Ka/Ks ratios, while all other genes had Ka/Ks ratios below 1, suggesting that they evolved under purifying selection. This collective evidence indicates that environmental factors exert relatively weak selection pressures on the genomes of *Strobilomyces* fungi.

Mitochondrial gene re-arrangements are common in fungi (Wu et al. 2021; Winkworth et al. 2022). Our analysis revealed structural re-arrangements in *Strobilomyces* species from various geographical regions. Through gene order comparisons, we identified two significant gene re-arrangements. Specifically, a reverse rearrangement involving the genes *trnG*, *atp9*, *trnD*, *trnW*, *trnK*, *trnQ*, *trnT*, *trnF*, *trnA*, *cox2* and *rnl* was observed in *Strobilomyces* species from the USA, mirroring patterns found in the mitogenomes of certain *Boletus* species (Li et al. 2021). Additionally, a short reverse re-arrangement affecting the genes *trnH*, *trnM* and *atp6* was identified in *Strobilomyces* species from Vietnam. Han et al. (2018) proposed that *Strobilomyces* species originated in Africa and dispersed to Southeast Asia via boreotropical forests during the early Eocene, with species in the Northern Hemisphere and Australasia primarily descending from Southeast Asian ancestors. Previous research has shown that the accumulation of repetitive sequences can trigger the recombination of fungal mitogenomes, significantly contributing to their re-arrangement (Winkworth et al. 2022). The observation of a significant number of repeats in the mitogenomes of *Strobilomyces* species suggests that these sequences may be a primary driver of the large-scale gene re-arrangements observed in these fungi. The cumulative effect of repeated evolutionary events in fungal mitogenomes often results in the extensive dispersal of repeat sequences, ultimately contributing to dynamic changes in genome structure and gene order (Winkworth et al. 2022).

Despite the ease of recognising *Strobilomyces* species by their distinctively coloured pileus covered with scales, the classification status of this genus within *Boletales* has been ambiguous (Han et al. 2020). With the advent of high-throughput sequencing technology, mitogenomes have become a valuable resource for phylogenetic analysis in plants, fungi and animals (Sun et al. 2021; Winkworth et al. 2022; Xue et al. 2024). In the field of fungal systematics, mitogenomes serve as a complementary resource to nuclear coding genes, which have traditionally been utilised in phylogenetic studies of *Boletales*. Mitochondria, essential organelles in eukaryotic cells, exhibit significantly greater complexity and diversity in fungal mitogenomes compared to those of animals, encompassing variations in size, repetitive content, functional genes and other structural attributes (Li et al. 2019). However, the limited availability of fungal mitogenomes has hindered in-depth studies on their evolution within *Boletales*. In this study, we re-affirmed the delimitation of *Strobilomyces* based on 14 core PCGs of the mitogenomes and nrDNA dataset, achieving relatively high bootstrap support despite some minor discrepancies. Our mitochondrial phylogenomic analysis validates the division of *Boletales* into seven major clades, aligning with established classification systems at the family level. Our findings are largely consistent with previous studies that relied on fragmentary DNA markers (Wu et al. 2014; Tremble et al. 2024).

## ﻿Conclusions

In this study, we have assembled and comprehensively characterised the mitogenomes of five *Strobilomyces* species. The sizes of these mitogenomic range from 35,618 bp to 42,088 bp, reflecting the inherent diversity within this genus. Through comparative analyses, we have identified both conserved and variable features of the *Strobilomyces* mitogenomes. Notably, we discovered two distinct patterns of gene re-arrangement in the mitogenomes of species originating from China, the USA and Vietnam. These findings provide valuable insights into the dynamics of fungal mitogenome evolution. Furthermore, the phylogenetic analysis, based on these mitogenomes, supports the classification of *Strobilomyces* within *Boletales*. This study not only enhances our understanding of the mitogenomes of fungi in *Boletales*, but also contributes important resources for elucidating broader evolutionary patterns of fungal mitogenomes.

## ﻿Additional information

### Adherence to national and international regulations

All the fungal strains used in this study have been legally obtained, respecting the Convention on Biological Diversity (Rio Convention).

### Author ORCIDs

Chao Liu https://orcid.org/0000-0001-6811-2218

Huan-Huan Chen https://orcid.org/0000-0001-9477-1014

Li-Hong Han https://orcid.org/0000-0002-6127-0915

### Data availability

All data used in this study are publicly available. The assembled mitogenome sequence of *S.alpinus* was submitted to the China National Center for Bioinformation with accession number C_AA098151.1 (https://ngdc.cncb.ac.cn/genbase/) and National Center for Biotechnology Information with accession number PQ568401 (https://www.ncbi.nlm.nih.gov).

## Supplementary materials

Supplementary material 1 Supplementary tables Data type xlsx Explanation note **table S1.** Species information used for constructing the phylogenetic trees; **table S2.** Number and types of SSRs in the five *Strobilomyces* mitogenomes; **table S3.** Number of interspersed repetitive sequences in the five *Strobilomyces* mitogenomes. https://binary.pensoft.net/file/1253229This dataset is made available under the Open Database License (http://opendatacommons.org/licenses/odbl/1.0/). The Open Database License (ODbL) is a license agreement intended to allow users to freely share, modify, and use this Dataset while maintaining this same freedom for others, provided that the original source and author(s) are credited.Chao Liu, Wan-Ying Li, Le-Xuan Zheng, Mi Dao, Huan-Huan Chen, Li-Hong Han

Supplementary material 2 Supplementary image Data type docx Explanation note **figure S1.** Amino acid frequency of 15 PCGs in the *S.alpinus* mitogenome. https://binary.pensoft.net/file/1253230This dataset is made available under the Open Database License (http://opendatacommons.org/licenses/odbl/1.0/). The Open Database License (ODbL) is a license agreement intended to allow users to freely share, modify, and use this Dataset while maintaining this same freedom for others, provided that the original source and author(s) are credited.Chao Liu, Wan-Ying Li, Le-Xuan Zheng, Mi Dao, Huan-Huan Chen, Li-Hong Han
