# Supplementary material for: ﻿Comparative mitogenomic analysis reveals variations and evolution of ectomycorrhizal fungal Strobilomyces
Source: IMA Fungus. 2025 Feb 17;16:e141848. doi: 10.3897/imafungus.16.141848 (PMC11882025; doi:10.3897/imafungus.16.141848)
Supplement: Supplementary material 2 — Supplementary image [file imafungus-16-e141848-s002.docx]

Figure S1 Amino acid frequency of 15 PCGs in the *S. alpinus* mitogenome.
